# Uropathogenic *Escherichia coli* Subverts Host Autophagic Defenses by Stalling Preautophagosomal Structures to Escape Lysosome Exocytosis

**DOI:** 10.1093/infdis/jiae063

**Published:** 2024-02-08

**Authors:** Xueping Li, Lingyan Jiang, Si Zhang, Jiarui Zhou, Le Liu, Chen Jin, Hongmin Sun, Qian Wang, Yutao Liu, Yu Pang

**Affiliations:** Tianjin Economic-Technological Development Area Institute of Biological Sciences and Biotechnology, Nankai University, Tianjin, People’s Republic of China; The Key Laboratory of Molecular Microbiology and Technology, Tianjin Economic-Technological Development Area Institute of Biological Sciences and Biotechnology, Nankai University, Ministry of Education, Tianjin, People’s Republic of China; Tianjin Economic-Technological Development Area Institute of Biological Sciences and Biotechnology, Nankai University, Tianjin, People’s Republic of China; The Key Laboratory of Molecular Microbiology and Technology, Tianjin Economic-Technological Development Area Institute of Biological Sciences and Biotechnology, Nankai University, Ministry of Education, Tianjin, People’s Republic of China; Tianjin Economic-Technological Development Area Institute of Biological Sciences and Biotechnology, Nankai University, Tianjin, People’s Republic of China; The Key Laboratory of Molecular Microbiology and Technology, Tianjin Economic-Technological Development Area Institute of Biological Sciences and Biotechnology, Nankai University, Ministry of Education, Tianjin, People’s Republic of China; School of Integrative Medicine, Tianjin University of Traditional Chinese Medicine, Tianjin, People’s Republic of China; Tianjin Economic-Technological Development Area Institute of Biological Sciences and Biotechnology, Nankai University, Tianjin, People’s Republic of China; The Key Laboratory of Molecular Microbiology and Technology, Tianjin Economic-Technological Development Area Institute of Biological Sciences and Biotechnology, Nankai University, Ministry of Education, Tianjin, People’s Republic of China; Tianjin Economic-Technological Development Area Institute of Biological Sciences and Biotechnology, Nankai University, Tianjin, People’s Republic of China; The Key Laboratory of Molecular Microbiology and Technology, Tianjin Economic-Technological Development Area Institute of Biological Sciences and Biotechnology, Nankai University, Ministry of Education, Tianjin, People’s Republic of China; Tianjin Economic-Technological Development Area Institute of Biological Sciences and Biotechnology, Nankai University, Tianjin, People’s Republic of China; The Key Laboratory of Molecular Microbiology and Technology, Tianjin Economic-Technological Development Area Institute of Biological Sciences and Biotechnology, Nankai University, Ministry of Education, Tianjin, People’s Republic of China; Tianjin Economic-Technological Development Area Institute of Biological Sciences and Biotechnology, Nankai University, Tianjin, People’s Republic of China; The Key Laboratory of Molecular Microbiology and Technology, Tianjin Economic-Technological Development Area Institute of Biological Sciences and Biotechnology, Nankai University, Ministry of Education, Tianjin, People’s Republic of China; Tianjin Economic-Technological Development Area Institute of Biological Sciences and Biotechnology, Nankai University, Tianjin, People’s Republic of China; The Key Laboratory of Molecular Microbiology and Technology, Tianjin Economic-Technological Development Area Institute of Biological Sciences and Biotechnology, Nankai University, Ministry of Education, Tianjin, People’s Republic of China; Tianjin Economic-Technological Development Area Institute of Biological Sciences and Biotechnology, Nankai University, Tianjin, People’s Republic of China; The Key Laboratory of Molecular Microbiology and Technology, Tianjin Economic-Technological Development Area Institute of Biological Sciences and Biotechnology, Nankai University, Ministry of Education, Tianjin, People’s Republic of China; Tianjin Economic-Technological Development Area Institute of Biological Sciences and Biotechnology, Nankai University, Tianjin, People’s Republic of China; The Key Laboratory of Molecular Microbiology and Technology, Tianjin Economic-Technological Development Area Institute of Biological Sciences and Biotechnology, Nankai University, Ministry of Education, Tianjin, People’s Republic of China

**Keywords:** uropathogenic *Escherichia coli*, PldA, autophagic flux, lysosome exocytosis

## Abstract

Urinary tract infections are primarily caused by uropathogenic *Escherichia coli* (UPEC). UPEC infects bladder epithelial cells (BECs) via fusiform vesicles and escapes into the cytosol by disrupting fusiform vesicle membrane using outer membrane phospholipase PldA, and establishes biofilm-like intracellular bacterial communities (IBCs) for protection from host immune clearance. Cytosolic UPEC is captured by autophagy to form autophagosomes, then transported to lysosomes, triggering the spontaneous exocytosis of lysosomes. The mechanism by which UPEC evades autophagy to recognize and form IBCs remains unclear. Here, we demonstrate that by inhibiting autophagic flux, UPEC PldA reduces the lysosome exocytosis of BECs. By reducing intracellular phosphatidylinositol 3-phosphate levels, UPEC PldA increases the accumulation of NDP52 granules and decreases the targeting of NDP52 to autophagy, hence stalling preautophagosome structures. Thus, our results uncover a critical role for PldA to inhibit autophagic flux, favoring UPEC escapes from lysosome exocytosis, thereby contributing to acute urinary tract infection.

## Teaser

This study uncovers a critical role of PldA through reducing intracellular PI3P levels to inhibit preautophagosomal structure maturation and autophagic flux, favoring UPEC escape from host lysosome exocytosis, thereby contributing to acute UTI.

Urinary tract infections (UTIs), affecting 150 million people each year globally, are one of the most common bacterial infections [1, 2]. Uropathogenic *Escherichia coli* (UPEC) is the main pathogen that causes 75%–95% of UTIs [3]. After the initial infection, recurrence occurs in 25% of patients within 6 months, with 68% of recurrent infections caused by the original strain, despite the use of appropriate antibiotic therapy [4, 5].

UPEC invades superficial bladder epithelial cells (BECs) by fusiform vesicles [6, 7]. UPEC utilizes outer membrane phospholipase PldA to disrupt fusiform vesicle membrane [8], escaping into BEC cytosol to evade toll-like receptor 4 (TLR4)-mediated exocytosis [9]. In the cytosol, UPEC replicates to form intracellular bacterial communities (IBCs), which protects UPEC from host innate immune defenses and antibiotic killing [10]. Cytosolic UPEC can be recognized and captured by autophagy to form autophagosomes. Autophagosomes containing UPEC fuse with multivesicular bodies and are transported to lysosomes, triggering the spontaneous exocytosis of lysosomes [11]. We have demonstrated that PldA facilitates UPEC escape from TLR4-mediated exocytosis by degrading fusiform vesicle membranes [8]. However, whether PldA facilitates UPEC escape from autophagosomes remains unknown.

Autophagy is a housekeeping process that maintains cellular homeostasis [12]. Autophagosome formation starts with the engulfment of cytosolic material by the phagophore, which then fuse with lysosomes to degrade the cargo [13]. Intracellular bacteria have evolved many strategies to cope with degradation by autophagy [14]. They antagonize autophagy initiation or autophagosomal maturation, evade autophagic recognition, or use components of the autophagy pathway to facilitate their intracellular survival [15–17]. However, whether UPEC can antagonize the autophagy pathway remains unknown.

Here, we showed that in addition to degrading fusiform vesicle membranes, PldA contributes to UPEC escape from lysosomal exocytosis. Through inhibiting autophagic flux and preautophagosomal structure maturation, PldA facilitates UPEC escape from host lysosome exocytosis, thereby contributing to acute UTI.

## METHODS

### Bacterial Strains

The strains and plasmids used in this study are listed in Supplementary Tables 1 and 2. UPEC strain CFT073 was utilized as the wild-type (WT) strain in this study. Δ*pldA* was generated using the λ-Red recombinase method [18]. The complemented strain Δ*pldA+* was generated by ligating *pldA* into the genomic DNA between *glmS* and *pstS* of Δ*pldA*.

### Bacterial Exocytosis Assay

For the bacterial exocytosis assay, 5637 cells (American Type Culture Collection HTB-9) were seeded into 24-well plates, pretreated with either vehicle (control group) or 2.5 mM 3-methyladenine (3-MA) for 12 hours, and infected with bacteria at a multiplicity of infection of 100. After 1 hour, cells were washed and treated with Roswell Park Memorial Institute (RPMI) 1640 medium containing 100 μg/mL gentamicin for 1 hour to kill all extracellular bacteria. Cells were then permeabilized with 0.1% Triton X-100 to determine the initial number of bacterial colony-forming units. The remaining monolayer was additionally cultured with 500 μL of fresh culture medium containing 100 mM methyl-D-mannopyranoside and the bacteriostatic reagent trimethoprim at 25 μg/mL for 4 hours. Then, 100 μL of the pooled culture supernatant was plated on Luria-Bertani (LB) agar plates. The percentage of extracellular bacteria was normalized against initial intracellular bacterial titer at 2 hours.

### Ex Vivo Bacterial Expulsion Assay

An ex vivo bacterial exocytosis assay was performed as described previously [19]. Six- to eight-week-old female BALB/c mice were transurethrally inoculated with 35 mM 3-MA or phosphate-buffered saline (PBS) for 12 hours. After inoculation of indicated strains for 1 hour, the bladder was emptied and injected with 100 μg/mL gentamycin in 50 μL of PBS for 1 hour to kill extracellular bacteria. After gentamicin treatment, the mice were sacrificed and bladders were collected. After PBS washing, bladder was cut open and incubated with 500 µL of Hanks’ balanced salt solution (HBSS) buffer at 37°C 5% CO_2_ for 6 hours. The exocytosed bacteria in the extracellular buffer were assessed by counting colonies plated on agar.

### Immunofluorescence Microscopy

Cells attached to coverslips were fixed with paraformaldehyde and permeabilized, and then incubated with the primary antibodies anti-LC3, human NDP52, SQSTM1/p62, or LAMP-1 and fluorophore-conjugated secondary antibodies. The coverslips were mounted in antifade reagent with 4′,6-diamidino-2-phenylindole (DAPI) for 5 minutes. To stain the bladder tissue, the bladder was fixed and blocked, followed by incubation with primary antibodies anti-mouse NDP52 and fluorescently conjugated secondary antibodies. The stained cells or bladders were imaged with a Zeiss LSM800 confocal microscope. In each case, 5 random fields were counted for each condition, in 3 independent experiments.

### Western Blotting

Samples were boiled in sodium dodecyl sulfate polyacrylamide gel electrophoresis (SDS-PAGE) loading buffer, and were separated by 12% SDS-PAGE and transferred to a polyvinylidene fluoride (PVDF) membrane. The membrane was blocked and probed with an anti-LC3 or anti-SQSTM1/P62 mouse monoclonal primary antibody overnight. The membranes were incubated with the secondary antibody. The proteins were visualized with enhanced chemiluminescence western blotting detection reagents using the ChemiDoc MP imaging system (Bio-Rad).

### Statistical Analyses

All experiments were repeated 3 times independently. In vitro results were analyzed with the unpaired Student *t* test, and in vivo results were analyzed with the Mann-Whitney *U* test. Differences for which *P* < .05 were considered statistically significant.

## RESULTS

### PldA Contributes to UPEC Escape From Lysosome Exocytosis of BECs

**Figure 1.**
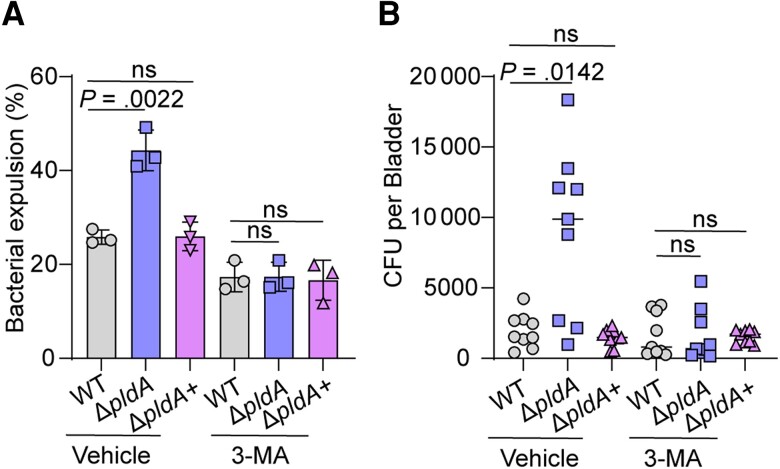
PldA contributes to UPEC escape from lysosomal exocytosis of BECs. *A*, Bacterial expulsion of WT, Δ*pldA,* or Δ*pldA*+ at 4 hours postinfection in infected 5637 cells pretreated with either vehicle or 2.5 mM 3-MA for 12 hours. *B*, Bacterial exocytosis into HBSS buffer after 6 hours of incubation with WT, Δ*pldA,* or Δ*pldA*+ infected mouse bladders pretreated with or without 3-MA, n = 9. Experiments were repeated 3 times. Data are presented as the mean ± SD. *P* values were determined using Student *t* test (*A*) and Mann-Whitney *U* test (*B*). Significance is indicated by the *P* value. Abbreviations: 3-MA, 3-methyladenine; BEC, bladder epithelial cells; CFU, colony-forming unit; HBSS, Hanks’ balanced salt solution; ns, not significant; UPEC, uropathogenic *Escherichia coli*; WT, wild type.

Previously, we established that PldA aids UPEC evasion of BEC exocytosis; the cell exocytosis experiment showed that the percentage of extracellular Δ*pldA* was significantly increased by 2.0-fold relative to that of WT at 6 hours postinfection (hpi) [8]. Combined with the conclusion from Miao et al that 6 hpi is the time point when most UPEC is exocytosed by lysosomes [11], we speculated that PldA may also participate in the escape of UPEC from lysosomes. The 5637 cells were pretreated with the autophagy inhibitor 3-MA or vehicle, and the invasion and exocytosis of 5637 cells infected with WT, Δ*pldA,* or Δ*pldA*+ were assayed at 4 hpi, which is the time point when the majority of UPEC undergo autophagy [11]. The number of Δ*pldA* that were exocytosed increased and the number that invaded decreased compared to that of WT or Δ*pldA*+ (Figure 1*A* and Supplementary Figure 1). Treatment with 3-MA resulted in reduced exocytosis in Δ*pldA*-infected 5637 cells to levels similar to those in WT or Δ*pldA*+ infected (Figure 1*A*). The number of Δ*pldA* that invaded 5637 cells increased significantly after 3-MA treatment compared with the control group, but was lower than that of WT or Δ*pldA*+ (Supplementary Figure 1). This result may be due to a defect in the bacteria to escape fusiform vesicles in the absence of PldA. Consistent with in vitro results, the number of exocytosed bacteria in Δ*pldA*-infected BALB/c mouse bladders increased compared to that of the WT or Δ*pldA*+ at 6 hpi. We also transurethrally treated mouse bladders with 3-MA to evaluate the function of PldA in autophagy in vivo. Firstly, we evaluated the effect of transurethral treatment with 35 mM 3-MA on the amount of LC3-II in mouse bladders. Transurethral treatment with 35 mM 3-MA for 12 hours before infection reduced the amount of LC3-II in mouse bladders (Supplementary Figure 2*A*), validating the inhibition effect of 3-MA in the autophagy process in vivo. Secondly, we enumerated the number of binucleated superficial BECs in mouse bladder with 3-MA or vehicle treatment. The number of superficial BECs was unaffected under 3-MA treatment (Supplementary Figure 2*B*), indicating the potentially cytotoxic effect of 3-MA did not affect the exfoliation of superficial BECs. Together, these results validate that 3-MA can be used as an autophagy inhibitor in vivo. Inhibitor-treated mouse bladders showed reduced exocytosis of Δ*pldA* to levels similar to those of WT or Δ*pldA*+ (Figure 1*B*). These data imply that PldA is required for UPEC to evade lysosome exocytosis both in vitro and ex vivo.

### PldA Contributes to UPEC Escape From Autolysosomes by Affecting the Autophagy Pathway

**Figure 2.**
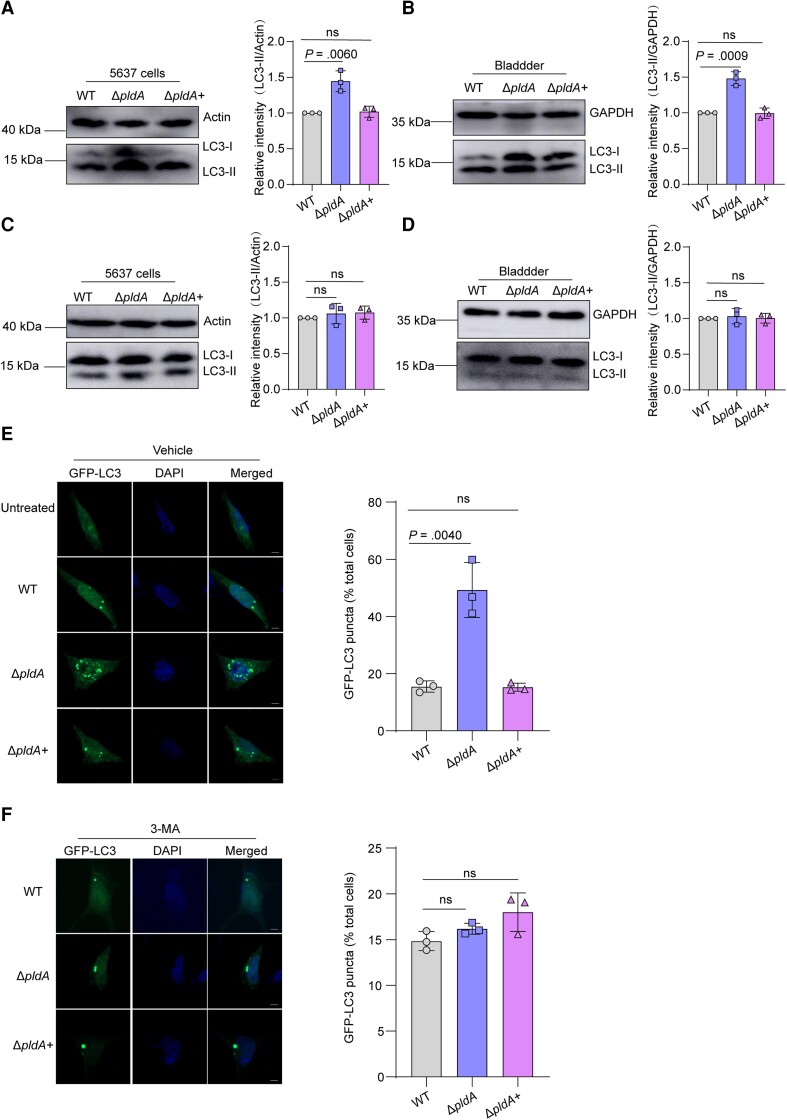
PldA contributes to UPEC escape from autolysosomes by affecting autophagy. *A*, Western blotting of LC3-II levels in WT, Δ*pldA,* or Δ*pldA*+ infected 5637 cells at 4 hpi. Actin was used as a loading control. *B*, Western blotting of LC3-II levels in WT, Δ*pldA,* or Δ*pldA*+ infected mouse bladders at 6 hpi. GAPDH was used as a loading control. *C*, Western blotting of LC3-II in WT, Δ*pldA,* or Δ*pldA*+ infected 5637 cells treated with 3-MA. The LC3-II signal densities were normalized to that of actin. *D*, Western blotting of LC3-II in WT, Δ*pldA,* or Δ*pldA*+ infected mouse bladders treated with 3-MA. The LC3-II signal densities were normalized to that of GAPDH. *E*, 5637 cells were transfected with GFP-LC3 for 24 hours and infected with the indicated CFT073 strains or left uninfected. The cells were analyzed for GFP-LC3 puncta. Representative confocal images are shown. The bar graph shows the quantification of LC3 puncta per cell. Scale bar, 5 μm. n = 3 slides. *F*, 5637 cells were transfected with GFP-LC3 for 24 hours, followed by treatment with 3-MA and infection with the indicated CFT073 strains. The cells treated with 3-MA were analyzed for GFP-LC3 puncta. Representative confocal images are shown. The bar graph shows the quantification of LC3 puncta per cell. Scale bar, 5 μm. n = 3 slides. Data are representative of 3 independent experiments and presented as the mean ± SD. The bar graph shows the relative LC3-II/actin (*A* and *C*) and LC3-II/GAPDH (*B* and *D*) densities. Data are presented as the mean ± SD. *P* values were determined using Student *t* test. Significance is indicated by the *P* value. Abbreviations: 3-MA, 3-methyladenine; DAPI, 4′,6-diamidino-2-phenylindole; GAPDH, glyceraldehyde-3-phosphate dehydrogenase; GFP, green fluorescent protein; hpi, hours postinfection; ns, not significant; UPEC, uropathogenic *Escherichia coli;* WT, wild type.

Because PldA is essential for UPEC to evade lysosome exocytosis and lysosome exocytosis begins with autophagy-mediated capture of cytosolic UPEC, we next investigated whether PldA is involved in the autophagy of UPEC-infected BECs. We first examined the amount of LC3-II, a lipidated form of LC3 used as a marker of autophagy [20], and P62, a marker of autophagic degradation [21], in UPEC-infected 5637 cells and mouse bladders. Δ*pldA*-infected 5637 cells showed increased amounts of LC3-II and decreased amounts of P62 compared to WT-infected 5637 cells and mouse bladders (Figure 2*A* and 2*B*, and Supplementary Figure 2*C* and 2*D*). These results indicate that PldA inhibits the autophagy pathway both in vitro and in vivo.

To confirm that PldA suppresses the activation of autophagy, 5637 cells and BALB/c mice were pretreated with 3-MA, and the amount of LC3-II in 5637 cells and mouse bladders infected with WT, Δ*pldA,* or Δ*pldA*+ was assayed. 3-MA treatment reduced the amounts of LC3-II in Δ*pldA*-infected 5637 cells and mouse bladders to levels similar to those in WT or Δ*pldA*+ infected (Figure 2*C* and 2*D*). The result showed that PldA inhibits activation of the autophagy pathway in host cells both in vitro and in vivo.

The number of LC3-positive puncta in UPEC-infected BECs reflects the level of autophagy activity [22]. We sought to visualize this activity using green fluorescent protein (GFP)-LC3 expressing BECs infected with different strains and examined the formation of LC3-positive puncta. LC3 appeared diffuse throughout the cytoplasm in uninfected 5637 cells, but LC3-positive puncta were readily detectable in WT-infected cells, and Δ*pldA-*infected cells accumulated more LC3-positive puncta than WT infected (Figure 2*E*). Treatment with 3-MA reduced LC3-positive puncta in Δ*pldA*-infected cells to levels comparable to WT or Δ*pldA*+ infected (Figure 2*F*). These results confirmed that PldA blocks the activation of autophagy.

### PldA Is Sufficient for UPEC to Evade Autophagy in the Cytosol

**Figure 3.**
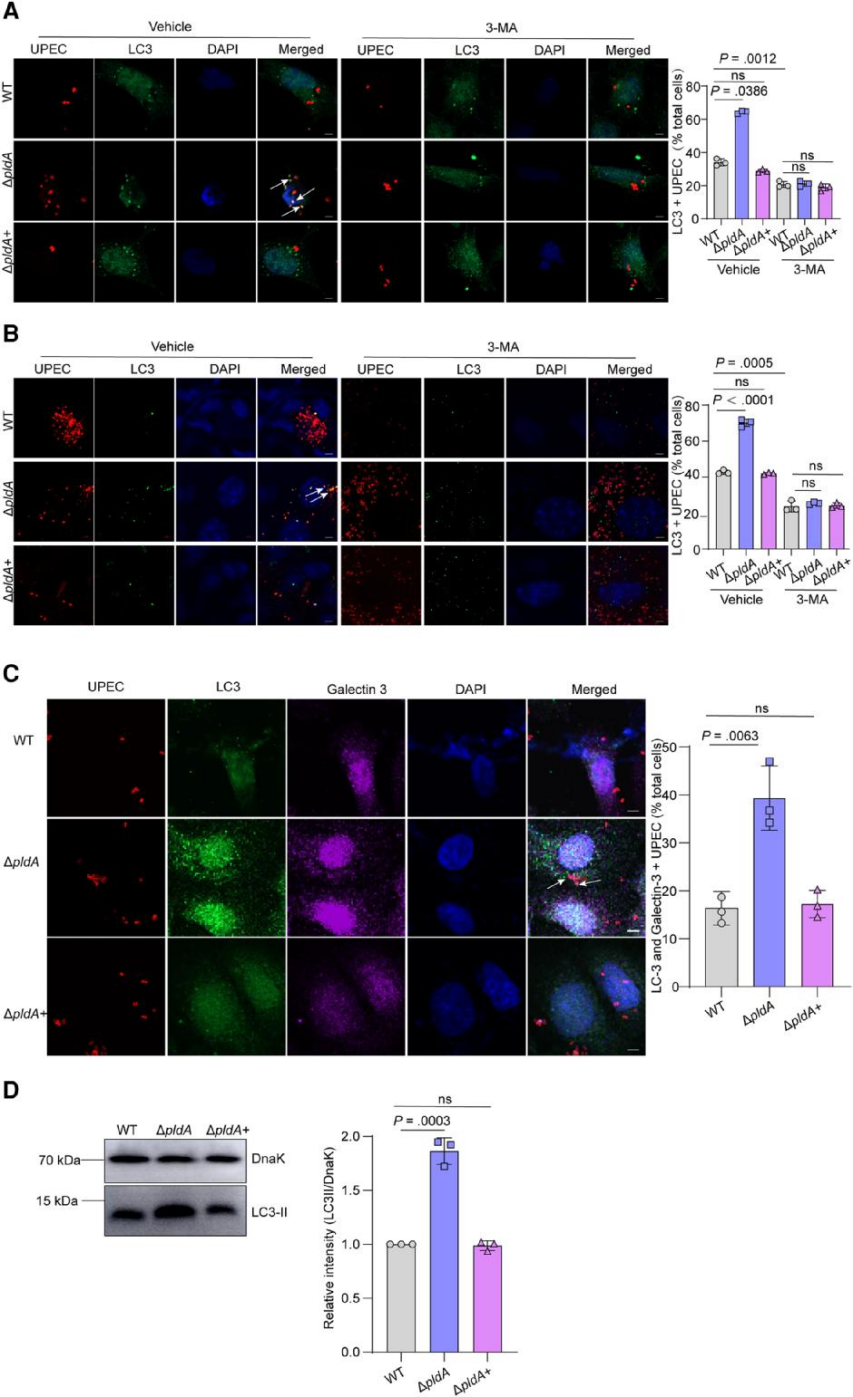
PldA is sufficient for autophagy evasion by UPEC in the cytosol. *A*, Immunofluorescence staining showing the colocalization of the autophagosome marker LC3 with the indicated strains expressing mCherry at 4 hours in 5637 cells pretreated with vehicle or 2.5 mM 3-MA for 12 hours. Representative confocal images are shown, and the arrow indicates LC3+ bacteria. The bar graph shows the percentage of intracellular bacteria that colocalized with LC3 relative to the total number of cells. Scale bar, 5 μm. n = 3 slides. *B*, Colocalization of LC3 with UPEC at 6 hpi in mouse bladders pretreated with vehicle or 35 mM 3-MA. Representative images are shown, and the arrow indicates LC3+ bacteria. The bar graph depicts the percentage of bacteria colocalizing with LC3 relative to the total number of cells. Scale bar, 5 μm. n = 3 slides. *C*, Colocalization of LC3 and galectin-3 with UPEC in 5637 cells at 4 hpi. Representative images are shown, and the arrow indicates LC3 and galectin-3+ bacteria. The bar graph depicts the percentage of bacteria colocalizing with LC3 and galectin-3 relative to the total number of cells. Scale bar, 5 μm. n = 3 slides. *D*, Western blotting of LC3-II in the vesicles containing bacteria from WT, Δ*pldA,* or Δ*pldA*+ infected 5637 cells. The LC3-II signal densities were normalized to that of bacteria DnaK. Data are presented as the mean ± SD. *P* values were determined using Student *t* test. Significance is indicated by the *P* value. Abbreviations: 3-MA, 3-methyladenine; DAPI, 4′,6-diamidino-2-phenylindole; hpi, hours postinfection; ns, not significant; UPEC, uropathogenic *Escherichia coli;* WT, wild type.

To ascertain whether PldA affects the capturing UPEC by autophagy, we evaluated the colocalization of LC3 or P62 with UPEC in vitro and in vivo. Δ*pldA* colocalized more than WT with LC3 or P62 in 5637 cells and mouse bladder (Figure 3*A* and 3*B*, and Supplementary Figure 3). Treatment with 3-MA reduced the colocalization of Δ*pldA* with LC3 to levels similar to WT or Δ*pldA*+ in 5637 cells and mouse bladders (Figure 3*A* and 3*B*), proving that PldA inhibits autophagy to capture intracellular UPEC.

UPEC undergoes a cytosolic period before captured by autophagy [23]. We next investigated whether PldA affected the capture of cytosolic UPEC by autophagy. Galectin-3 is a marker of vacuole lysis, and also plays an essential role in pathogen recognition in autophagosome formation. Intracellular bacteria that colocalized with both galectin-3 and LC3 indicates the bacteria that escaped into the cytosol are captured by autophagy [24, 25]. The colocalization of Δ*pldA* with both galectin-3 and LC3 was higher than that of WT in 5637 cells (Figure 3*C*), confirming PldA inhibits autophagy to capture the cytosolic UPEC.

Furthermore, we purified bacteria-containing vesicles in UPEC-infected 5637 cells and quantified the LC3 amount in isolated vesicles. UPEC were pretagged with magnetic nanoparticles before they were employed for 5637 cell infection, to isolate intracellular bacteria along with the vesicles using a magnet following a previously described method [8, 19, 26]. Western blotting result showed a 0.87-fold increase of LC3-II in the isolated fraction from Δ*pldA*-infected 5637 cells compared to WT infected (Figure 3*D*), indicating PldA inhibits autophagosome formation.

### PldA Induces the Accumulation of NDP52 Granules Through Reducing Intracellular PI3P Levels

**Figure 4.**
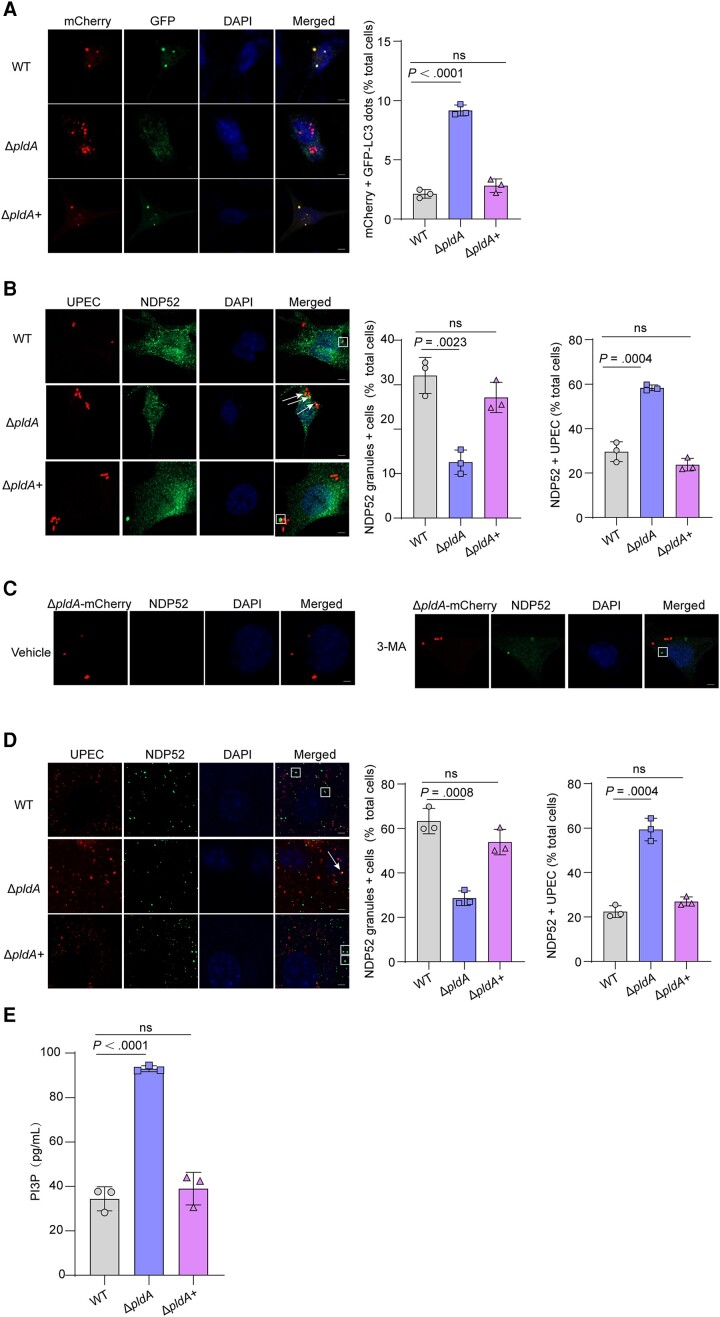
PldA induces the accumulation of NDP52 granules. *A*, Representative images of 5637 cells transfected with mCherry-GFP-LC3 for 24 hours and infected with WT, Δ*pldA* or Δ*pldA+* for 4 hours. Autophagosomes, yellow puncta; autolysosomes, red puncta. The bar graph shows the quantification of LC3 red puncta per cell. Scale bar, 5 μm. n = 3 slides. *B*, Colocalization of NDP52 with CFT073 expressing mCherry in 5637 cells at 4 hpi. The arrows indicate NDP52+ bacteria, and the square frames indicates NDP52 granules (left). The bar graph shows the percentage of cells infected with the indicated strains displaying 1 or several NDP52 granules (middle) and the intracellular bacteria that colocalized with NDP52 relative to the total number of cells (right). Scale bar, 5 μm. n = 3 slides. *C*, NDP52 granules of Δ*pldA-*mCherry–infected 5637 cells treated with either vehicle or 2.5 mM 3-MA. The square frame indicates an NDP52 granule. Scale bar, 5 μm. n = 3 slides. *D*, Colocalization of NDP52 with CFT073 expressing mCherry in mouse bladders at 6 hpi. The arrow indicates NDP52+ bacteria, and the square frame indicates NDP52 granules (left). The bar graph shows the percentage of cells infected with the indicated strains displaying 1 or several NDP52 granules (middle) and the intracellular bacteria that colocalized with NDP52 relative to the total number of cells (right). Scale bar, 5 μm. n = 3 slides. *E*, 5637 cells infected with WT, Δ*pldA,* or Δ*pldA+* for 4 hours were lysed and PI3P levels were measured by competitive ELISA. Data are presented as the mean ± SD. *P* values were determined using Student *t* test. Significance is indicated by the *P* value. Abbreviations: 3-MA, 3-methyladenine; DAPI, 4′,6-diamidino-2-phenylindole; ELISA, enzyme-linked immunosorbent assay; hpi, hours postinfection; ns, not significant; PI3P, phosphatidylinositol 3-phosphate; RFP-GFP, red fluorescent protein-green fluorescent protein; WT, wild type.

To fully understand PldA's role in the autophagy pathway, autophagic flux was measured. Cells were transfected with an expression vector encoding LC3 fused to GFP and red fluorescent protein (RFP) in tandem. This construct can be used to quantify the rate of progression from autophagosomes (yellow) to autolysosomes (red) as a measure of autophagic flux because GFP fluorescence is more sensitive to pH than RFP fluorescence and is quenched at lysosomal pH. Autophagic flux was significantly stronger in Δ*pldA*-infected 5637 cells than in WT infected, suggesting that PldA inhibits autophagic flux (Figure 4*A*).

We next investigated whether the observed reduction in autophagic flux caused by PldA resulted from reduced autophagosome formation. NDP52, which contains an LC3-interacting motif and a ubiquitin-binding domain, recruits the autophagy machinery to create a phagophore surrounding intracellular bacteria [27]. NDP52 granules that form in the vicinity but not directly in contact with intracellular bacteria represent a cytosolic preautophagosomal structure. WT-infected 5637 cells accumulated substantial amounts of cytosolic NDP52 granules (Figure 4*B*) and the accumulation of NDP52 granules was reduced in Δ*pldA*-infected 5637 cells, indicating that PldA was required for the accumulation of cytosolic NDP52 granules in the vicinity of intracellular UPEC. The percent colocalization of Δ*pldA* with NDP52 was increased compared with that of WT in 5637 cells (Figure 4*B*), indicating that PldA inhibits the recruitment of NDP52 to intracellular UPEC and the decoration of intracellular UPEC with NDP52 is indicative of autophagic targeting [28]. In addition, 3-MA treatment enhanced the reduced accumulation of cytosolic NDP52 granules in Δ*pldA*-infected 5637 cells compared to the control group (Figure 4*C*). In vivo, Δ*pldA* also accumulated fewer cytosolic NDP52 granules and decorated more with NDP52 than in WT-infected mouse bladders (Figure 4*D*). Combined with the result that PldA accumulates cytosolic NDP52 granules and reduces the colocalization of intracellular UPEC with NDP52, these results indicate that PldA stalls preautophagosomal formation.

We next aimed to investigate the mechanism through which PldA contributes to the accumulation of preautophagosomal structure. Phosphatidylinositol 3-phosphate (PI3P) which is generated from phosphatidylinositol (PI) by phosphatidylinositol 3-kinase (PI3K), plays an essential role in preautophagosomal structure formation [29]. *Listeria* phospholipases PlcA/B consumes PI to decrease intracellular PI3P levels, causing preautophagosomal structure stalling [28]. We speculate that UPEC PldA employs a strategy similar to the use of PlcA/B in *Listeria*. The levels of cellular PI3P infection with Δ*pldA* were dramatically higher than that of WT (Figure 4*E*), indicating that PldA reduces intracellular PI3P levels. These results suggest that by reducing intracellular PI3P levels, PldA contributes to the accumulation of preautophagosomal structures.

We next investigated whether PldA affects the autophagosome-lysosome fusion in host cells [30]. The colocalization of Δ*pldA* with lysosome marker LAMP-1 was higher than that of WT or Δ*pldA*+ in 5637 cells (Supplementary Figure 4*A* and 4*B*), indicating PldA inhibits the formation of UPEC-containing autolysosomes. Treatment of 5637 cells with autophagosome-lysosome fusion inhibitor bafilomycin A1 did not affect the percentage of colonization of Δ*pldA* with LAMP-1 (Supplementary Figure 4*A* and 4*B*), indicating PldA did not affect autophagosome-lysosome fusion. Also, we investigated the impact of PldA on lysosomal activity by measuring acid phosphatase activity in the culture medium. Deletion of *pldA* did not affect the acid phosphatase activity in UPEC-infected 5637 cells (Supplementary Figure 4*C*), indicating PldA did not influence host cell lysosomal activity. These results excluded the possibility that the reduced autophagic flux caused by PldA is due to decreased autophagosome-lysosome fusion.

### Inhibition of Preautophagosomal Maturation by PldA Protects UPEC Against Autophagy in Host Cells

**Figure 5.**
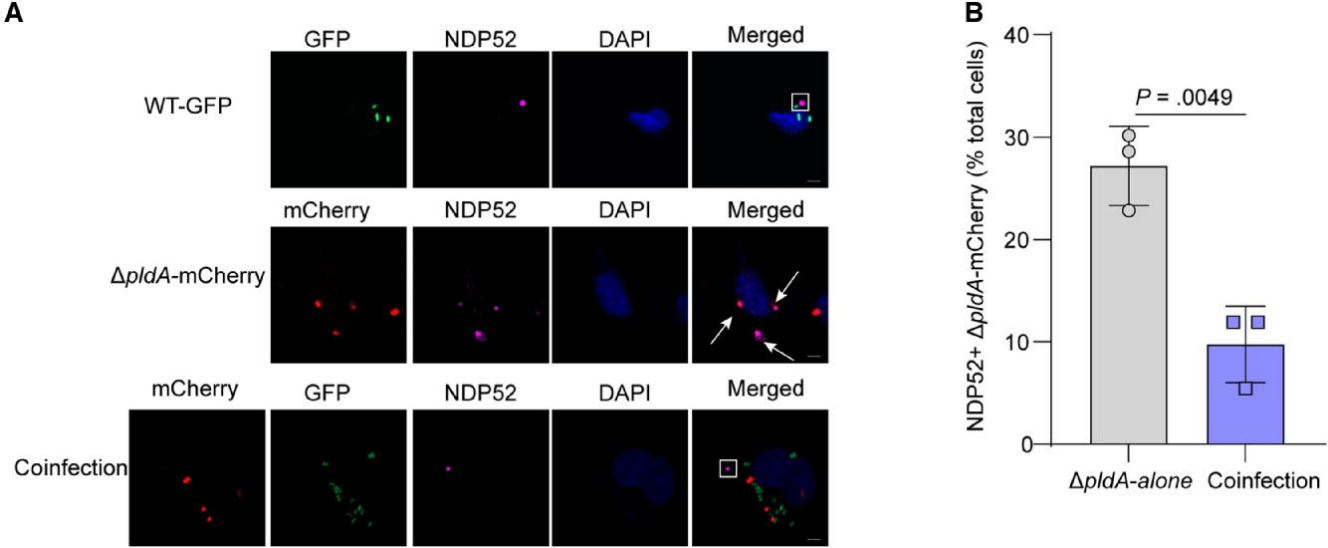
Inhibition of preautophagosomal maturation by UPEC PldA protects against autophagy in host cells. *A*, Colocalization of NDP52 with the WT strain expressing GFP, Δ*pldA* expressing mCherry, or both strains at a 1:1 ratio in 5637 cells at 4 hpi. The arrow indicates NDP52+ bacteria, and the square frame indicates NDP52 granules. Scale bar, 5 μm. *B*, Quantification of the intracellular bacteria that colocalized with NDP52 relative to the total number of cells infected with either Δ*pldA*-mCherry alone or WT-GFP and Δ*pldA*-mCherry (coinfection). n = 3 slides. Data are presented as the mean ± SD. *P* values were determined using Student *t* test. Significance is indicated by the *P* value. Abbreviations: DAPI, 4′,6-diamidino-2-phenylindole; GFP, green fluorescent protein; hpi, hours postinfection; ns, not significant; UPEC, uropathogenic *Escherichia coli*; WT, wild type.

To determine whether the capacity of PldA to stall preautophagosomal structures is responsible for UPEC inhibiting the host autophagy pathway, we used WT expressing GFP (WT-GFP) and Δ*pldA* expressing mCherry (Δ*pldA*-mCherry) in coinfection experiments. As controls, 5637 cells infected with WT-GFP alone showed the formation of cytosolic NDP52 granules (Figure 5*A*). Infection with Δ*pldA*-mCherry alone reduced cytosolic NDP52 granules formation, while numerous Δ*pldA*-mCherry colocalized with NDP52, indicative of autophagic targeting (Figure 5*A*). When 5637 cells were infected with both WT-GFP and Δ*pldA*-mCherry, the percent colocalization of Δ*pldA*-mCherry with NDP52 was significantly reduced (Figure 5*B*), suggesting that PldA produced by WT-GFP protected Δ*pldA*-mCherry from autophagic targeting. Taken together, we found that PldA-dependent inhibition of preautophagosomal structure maturation results in the formation of NDP52 granules in the cytosol of infected cells, contributing to protection of UPEC against autophagic targeting.

### PldA Contributes to UPEC Colonization During the Early Stage of Infection

**Figure 6.**
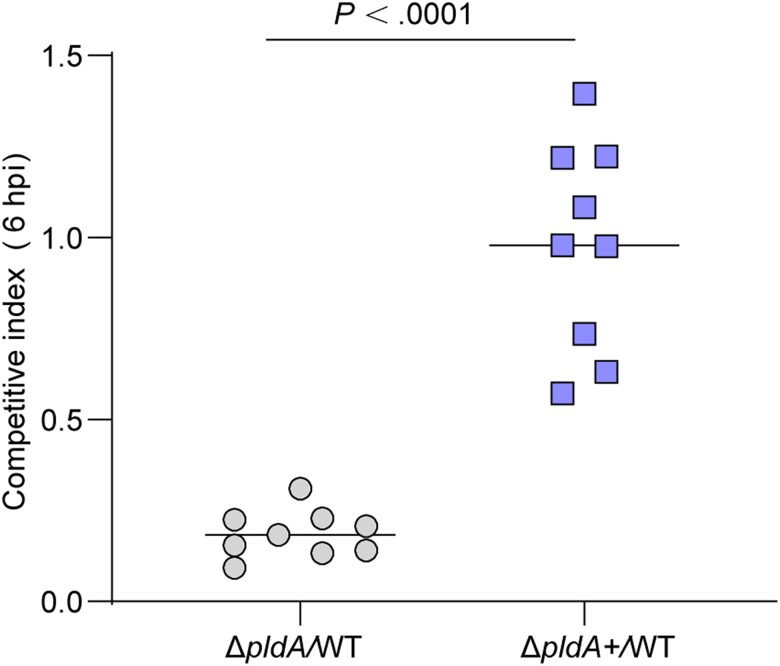
PldA contributes to UPEC colonization during the early stage of infection. Competitive indexes for Δ*pldA* or Δ*pldA+* versus WT in mouse bladders at 6 hpi. n = 9. Data were obtained from 3 independent experiments and are presented as the mean ± SD. *P* values were determined using the Mann-Whitney *U* test. Significance is indicated by the *P* value. Abbreviations: hpi, hours postinfection; UPEC, uropathogenic *Escherichia coli*; WT, wild type.

To confirm that PldA-mediated inhibition of autophagy contributes to UPEC virulence, we conducted competitive infection using Δ*pldA* or Δ*pldA+* with WT. The result showed that Δ*pldA* was significantly out competed at 6 hpi. However, equivalent bacterial loads were recovered for WT and Δ*pldA+* (Figure 6). Δ*pldA* grew as well as WT in LB and RPMI 1640 medium (Supplementary Figure 5), indicating that the decreased fitness of Δ*pldA* in vivo was not due to a growth defect. These results indicated that PldA contributes to UPEC virulence in the early stage of infection (IBC formation) [8]. Combined with the role of UPEC PldA in disrupting fusiform vesicles, the reduced colonization of Δ*pldA* may due to the dual functions of PldA in escaping fusiform vesicles and lysosome exocytosis.

## DISCUSSION

Expulsion of UPEC from BECs is a powerful cell-autonomous defense mechanism to rapidly reduce the intracellular bacterial load [31]. UPEC is expelled from BECs by cyclic AMP-mediated TLR4 signal pathway, triggering the expulsion of fusiform vesicles [9]. Our previous study found that PldA disrupts fusiform vesicles by degrading the membrane of these vesicles and facilitating UPEC escape in the host cell cytosol to form IBCs and cause UTIs [8]. Once UPEC escapes into the cytosol and evades the first wave of exocytosis, cytosolic UPEC is recognized and captured by autophagy [11, 23]. Canonical autophagy leads to the degradation of bacteria, but UPEC uses HlyA to defragment cytosolic microtubules to block lysosome acidification [23] and survive within lysosomes, triggering the exocytosis of lysosomes [11]. Exocytosis of lysosomal contents is an innate homeostatic mechanism of BECs, to remove cargos that have not been degraded. Here, we demonstrate that UPEC utilizes PldA to evade this powerful exocytosis mechanism by blocking autophagosome formation. The inhibition of autophagy caused by PldA reduces the maturation of autolysosomes, hence reducing the expulsion of UPEC by lysosomes. We found that the outer membrane phospholipase PldA, which blocks autophagosome formation in coordination with its capacity to degrade eukaryotic membranes, helps UPEC escape from 2 waves of host cell-autonomous defense mechanisms and contributes to bacterial colonization during the early stage of infection.

Autophagy has emerged as a critical cellular process for defense against intracellular bacterial pathogens [32]. Intracellular bacterial pathogens have evolved many strategies to subvert host autophagy. *Salmonella* Typhimurium secretes effector SopF to block host cell autophagy by blocking V-ATPase–recruited ATG16L1 onto bacteria-containing vacuoles [22]. *Shigella* uses effector IcsB to target a component of the ESCRT-III complex, specifically enabling *Shigella* escape from host cell autophagy [33]. *Listeria* phospholipases PlcA/B reduce PI3P levels by cleaving PI into diacylglycerol, resulting in reduced autophagic flux and causing preautophagosomal structure stalling to favor bacterial escape from host autophagic defense [28]. PldA, as an outer membrane phospholipase, can degrade a wide variety of phospholipids as well as PI. Our results showed that the levels of cellular PI3P were dramatically lower in the presence of PldA and 3-MA (which is an inhibitor of PI3K), and the reduced accumulation of NDP52 granules in Δ*pldA*-infected 5637 cells was enhanced compared to the control group. The results indicated a role of PldA in the consumption of PI to decrease intracellular PI3P levels.

PI3P is a regulator mainly affecting cellular membrane trafficking. The interaction between PI3P and target proteins is mediated by a conserved zinc-finger domain, FYVE [34]. The prototypic FYVE protein is EEA1, which is the early endosomal autoantigen that mediates early endosome fusion [35]. We speculate that the function of PldA to reduce PI3P levels may also affect early endosome fusion, which remains to be investigated in future studies. PI3P on the membrane serves as a recruitment signal to recruit autophagy-related protein, such as WIPI, which is required for phagophore maturation. NDP52 is a selective autophagy receptor that recognizes invading bacteria and interacts with LC3 on the autophagosome membrane, facilitating the engulfment of the bacteria into the autophagosome [36]. The function of PI3P to affect autophagy is prior to NDP52 activity; when PI3P falls under a threshold level the preautophagosomal structure is stalled, blocking the recruitment of NDP52 to undergo autophagy, leading to an accumulation of NDP52 granules [28]. The potential mechanism of PldA to increase NDP52 granules may due to its function in reducing intracellular PI3P levels. LC3-II is essential for elongation and closure of the autophagosome membrane. Therefore, any change in the levels of LC3-II can directly affect the efficiency and extent of autophagosome formation. Even a small increase or decrease in LC3-II levels can have a significant impact on the overall autophagic activity, influencing the degradation of cellular components and the regulation of cellular homeostasis [37]. This may explain how our western blotting result that the levels of LC3-II in Δ*pldA-*infected BECs, which increased about 0.5-fold compared to WT, can influence cellular physiology.

Autophagy acts as a double-edged sword in UPEC infection. Intracellular UPEC may be under the threat of autophagy clearance. Autolysosome-mediated exocytosis is a strategy that host cells use to defend against invading UPEC in the early stage of infection [11]. On the other hand, Wang and colleagues have found that UPEC exploits host ferritinophagy to persist within autophagosomes as quiescent intracellular reservoirs (QIRs) that can seed recurrent UTIs at the late stage of infection [38]. Intracellular UPEC traffics, together with ferritin-bound iron, into the autophagosome to form QIRs [39]. Our results reveal that UPEC utilizes PldA to block the activation of autophagy in the early stage of infection. Combined with the work of the Mysorekar group, the process by which UPEC interferes with autophagy during early and late stages of infection has been mostly illustrated.

In summary, our results show that UPEC PldA contributes to bacterial escape from autophagy by inhibiting autophagic flux and stalling maturing preautophagosomal structures. This finding provides new insights into the mechanisms that govern autophagy induction and maturation during infection with intracellular bacterial pathogens. Given the important role of PldA in acute UTI, it may be an ideal target for vaccine development for the treatment of UTIs.

## Supplementary Data

Supplementary materials are available at *The Journal of Infectious Diseases* online (http://jid.oxfordjournals.org/). Supplementary materials consist of data provided by the author that are published to benefit the reader. The posted materials are not copyedited. The contents of all supplementary data are the sole responsibility of the authors. Questions or messages regarding errors should be addressed to the author.

## Supplementary Material

jiae063_Supplementary_Data

## Notes

***Author contributions.*** Y. P. and X. L. performed study concept and design. X. P performed the research with the assistance of J. Z, L. L, H. S, Q. W, and S. Z. L. J. and Y. L. supervised the research and analyzed data. Y. P. and X. L. wrote the manuscript. All authors had the opportunity to discuss the results and comment on the manuscript.

***Acknowledgments.*** The authors thank Professor Tao Wang from School of Life Sciences, Tianjin University for sharing the GFP-LC3 plasmid.

***Financial support*.** This work was supported by the China Postdoctoral Science Foundation (grant number 2020M670623 to Y. P.); National Natural Science Foundation of China Program (grant numbers 32100144 to Y. L. and 32170110 to L. J.); and the Natural Science Foundation of Tianjin (grant number 22JCYBJC01060 to L. J.).
