# Supplementary material for: Uropathogenic Escherichia coli Subverts Host Autophagic Defenses by Stalling Preautophagosomal Structures to Escape Lysosome Exocytosis
Source: J Infect Dis. 2024 Feb 8;230(3):e548–58. doi: 10.1093/infdis/jiae063 (PMC11420784; doi:10.1093/infdis/jiae063)
Supplement: jiae063_Supplementary_Data [file jiae063_supplementary_data.zip › Supplementary_materials.docx]

Supplementary materials

Uropathogenic *Escherichia coli* subverts host autophagic defenses by stalling pre-autophagosomal structures to escape lysosome exocytosis

Xueping Li, Lingyan Jiang, Si Zhang, Jiarui Zhou, Le Liu, Chen Jin, Hongmin Sun, Qian Wang, Yutao Liu, Yu Pang

Contents:

5 Supplementary Figures Legends

2 Supplementary Tables

Supplementary materials and methods

**Supplementary Figure 1.** Bacterial titers of WT, Δ*pldA*, or Δ*pldA*+ at 4 h p.i in 5637 cells treated with vehicle or 2.5 mM 3-MA. 5637 cells were treated with vehicle or 3-MA overnight and then were infected with the indicated strains for 2 h, followed by an additional 2 h of incubation in medium containing 100 μg/mL gentamicin. Data are represented as mean ± SD. *P* values were determined using Student’s *t*-test. Significance is indicated as the P value. ns, no significant difference.

**Supplementary Figure 2.** (A) Western blotting of LC3-II levels in Δ*pldA*-infected mouse bladders pretreated with vehicle or 35 mM 3-MA for 12 h. GAPDH was used as a loading control. (B) BALB/c mice were treated with vehicle or 35 mM 3-MA for 12 h. After treatment, bladders were removed and fixed. Whole-mounted bladders were stained with DAPI and imaged by confocal microscopy. Representative confocal images are shown, and the bar graph shows the number of binucleate cells from five random fields of view. Scale bar, 20 μm. n = 3 slides. (C) Western blotting of P62 levels in WT-, Δ*pldA*- or Δ*pldA*+-infected 5637 cells at 4 h p.i. GAPDH was used as a loading control. (D) Western blotting of P62 levels in WT-, Δ*pldA*- or Δ*pldA*+-infected BALB/c bladders at 6 h p.i. Actin was used as a loading control. Data are represented as mean ± SD. *P* values were determined using Student’s *t*-test. Significance is indicated as the P value. ns, no significant difference.

**Supplementary Figure 3. PldA prevents autophagy from capturing intracellular UPEC.** Immunofluorescence staining showing the colocalization of P62 (green) with the indicated strains expressing mCherry (red) in 5637 cells at 4 h p.i. (A) and BALB/c bladders at 6 h p.i.(B). Representative confocal images are shown, and the bar graph shows the percentage of intracellular bacteria that colocalized with P62 relative to the total number of cells. Scale bar, 5 μm. n = 3 slides.

**Supplementary Figure 4. PldA inhibits the formation of UPEC-containing autolysosomes.** (A) Immunofluorescence staining of colocalization of LAMP-1 (green) with the indicated strains expressing mCherry (red) at 4 h p.i., in 5637 cells pre-treated with vehicle or 1 µM bafilomycin A1 for 12 h. Representative confocal images are shown, Scale bar, 5 µm. n = 3 slides. (B) Bar graph shows the percentage of intracellular bacteria that are colocalized with LAMP-1 relative to total cells. (C) Extracellular acid phosphatase activity in 5637 cells infected with UPEC strains at 4 h p.i. Data are represented as mean ± SD. *P* values were determined using Student’s *t*-test. Significance is indicated as the P value. ns, no significant difference.

**Supplementary Figure 5. Growth curves for UPEC CFT073 WT, Δ*pldA*, or Δ*pldA*+ in LB medium (A) and RPMI 1640 medium (B).** The absorbance of bacterial suspensions at 600 nm (OD_600_) was measured regularly using a microplate reader over a 24-hour time. Data were obtained from three independent experiments and are presented as mean ± SD.

Supplemental Table 1. Bacterial strains were used in this study.

| **Strain** | **Characteristics or function** | **Source/ Reference** |
| --- | --- | --- |
| *E. coli* CFT073 | An isolate from a patient with pyelonephritis | ATCC |
| Δ*pldA* | CFT073 Δ*pldA::*Cm; Cm^r^ | This study |
| Δ*pldA*+ | CFT073 Δ*pldA::pldA*, Cm, Km; Cm^r^, Km^r^ | This study |
| CFT073-mCherry | CFT073 containing pETDuet expressing *mcherry* gene encoding red fluorescence; Amp^r^ | This study |
| Δ*pldA-*mCherry | Δ*pldA* containing pETDuet expressing *mcherry* gene encoding red fluorescence; Cm^r^, Amp^r^ | This study |
| Δ*pldA+-*mCherry | Δ*pldA*+ containing pETDuet expressing *mcherry* gene encoding red fluorescence; Cm^r^, Amp^r^ | This study |

Supplemental Table 2. Plasmids were used in this study.

| **Name** | **Description** | **Source/Reference** |
| --- | --- | --- |
| pKD3 | Containing a chloramphenicol resistance cassette and the flipase recognition sites; Cm^r^ | This study |
| pSim | Red recombination plasmid; Bla^r^ | This study |
| RFP-GFP-LC3 | For overexpressing mCherry- and GFP-fused LC3 | MiaoLing Plasmid Sharing Platform |
| GFP-LC3 | For over-expressing GFP-fused LC3 | Prof. Wang's lab |
| pETDuet-mCherry | pETDuet harboring *mcherry* gene encoding red fluorescence protein; Amp^r^ | This study |

**Supplementary materials and methods**

**Reagents and antibodies**

For western blotting and immunofluorescence analyses, the following antibodies were used: anti-LC3 (MBL; M186-3), anti-human NDP52 (Abcam; ab68588), anti-mouse NDP52 (ABclonal; A22088), anti-SQSTM1/p62 (Abcam; ab109012), anti-LAMP-1 (Santa Cruz Biotechnology; sc-17768), anti-GAPDH (CST; 2118), anti-galectin 3 (Abcam; ab2785), anti-actin (Abcam; ab8226), goat anti-rabbit IgG (HRP, Abcam; ab6721), goat anti-mouse IgG (HRP; Abcam; ab6789), goat anti-mouse IgG FITC (Abcam; ab6785), and goat anti-rabbit IgG Alexa Fluor 647 (Abcam, ab150079).

Additional reagents include 3-methyladenine (Sigma; 189490), methyl D-mannopyranoside (Sigma; 462711), bafilomycin A1(LC Laboratories; B-1080), enhanced chemiluminescence (ECL) Western Blotting Detection Reagents (Thermo Fisher Scientific; 34580) and DAPI (Abcam; ab104139).

**Mice infection**

All animal studies were conducted according to protocols approved by the Institutional Animal Care Committee of Nankai University (Tianjin, China) and performed under protocol no. IACUC 2020030501. For competitive infection, 6-8-week-old female BALB/c mice were anesthetized with 3% chloral hydrate (Sangon Biotech; A600288) and transurethrally cochallenged with the inoculum of 1×10^7^ bacteria of Δ*pldA* and WT, or Δ*pldA*+ and WT. At 6 h p.i., mice were sacrificed, and bladders were harvested aseptically. The bladder was treated with 100 μg/mL gentamicin sulfates for an additional 30 min to kill extracellular bacteria. Then, the bladder was aseptically removed and homogenized in 1 mL of sterile PBS. Homogenates were serially diluted with PBS and cultured on LB agar plates for CFU determination. The competitive index was calculated as the ratio of recovered CFU of Δ*pldA* or Δ*pldA* + mutant/recovered CFU of WT*.*

**Isolation of intracellular vesicles containing bacteria**

To isolate intracellular vesicles containing bacteria, UPECs were conjugated with BioMag carboxyl magnetite nanoparticles (BM570; Bangs Laboratories) to generate ‘‘magnetic bacteria’’. The magnetite nanoparticles were washed twice in 0.1 MMES buffer (2-(N-morpholino) ethanesulphonic acid), and were activated with 40 mg EDAC (1-ethyl-3-(3-dimethylaminopropyl) carbodiimide). The activated magnetite nanoparticles were mixed with 5 × 10^9^ live UPEC strains and incubated for 30 min at 37℃. After blocking with 1% BSA, the magnetic bacteria were added to 5637 cells at an MOI of 100. After infection, cells were scrapped and the plasma membrane was disrupted by repeated passage of the cell suspension through a 29-gauge needle (BD, 328421). The debris of host cells were removed by centrifugation at 2,000 × g at 4℃ for 5 min. The vesicles containing bacteria in the supernatant were harvested at 4℃ using a magnetic cell separation rack (Invitrogen), then washed with cold PBS twice. The amount of LC3-II in vesicles containing bacteria was analyzed after SDS-PAGE and western blotting.

**Lysosome enzyme release/activity**

The 5637 cells were infected with bacteria for 4 h. Acid phosphatase (AP) activity in the culture medium was measured using AP colorimetric assay kit (Beyotime; P0326) according to the manufacturer’s instruction. Briefly, the substrate p-nitrophenyl phosphate (pNPP) was mixed with BEC culture medium and dephosphorylated by AP. The amount of the dephosphorylated pNPP was measure at a wavelength of 405nm for AP activity.
