# Supplementary figures and images for: Uropathogenic Escherichia coli Subverts Host Autophagic Defenses by Stalling Preautophagosomal Structures to Escape Lysosome Exocytosis
Source: J Infect Dis. 2024 Feb 8;230(3):e548–58. doi: 10.1093/infdis/jiae063 (PMC11420784; doi:10.1093/infdis/jiae063)

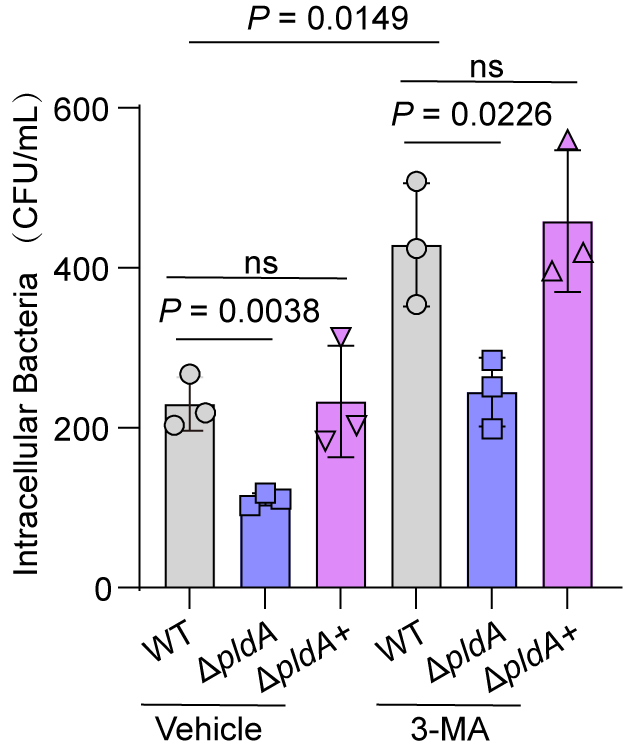

Supplement: jiae063_Supplementary_Data [file jiae063_supplementary_data.zip › SupplementaryFigure 1.tif]

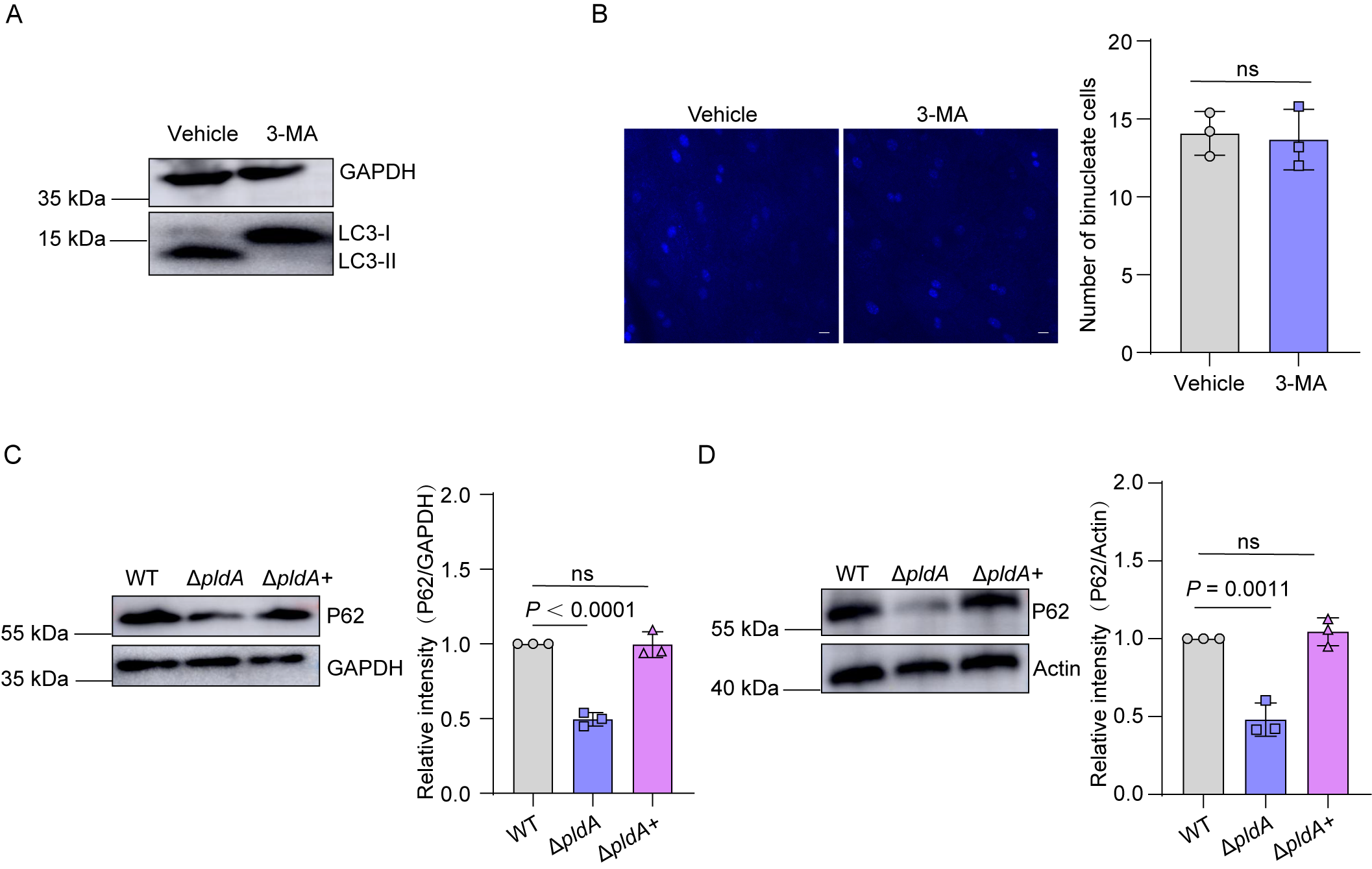

Supplement: jiae063_Supplementary_Data [file jiae063_supplementary_data.zip › SupplementaryFigure 2.tif]

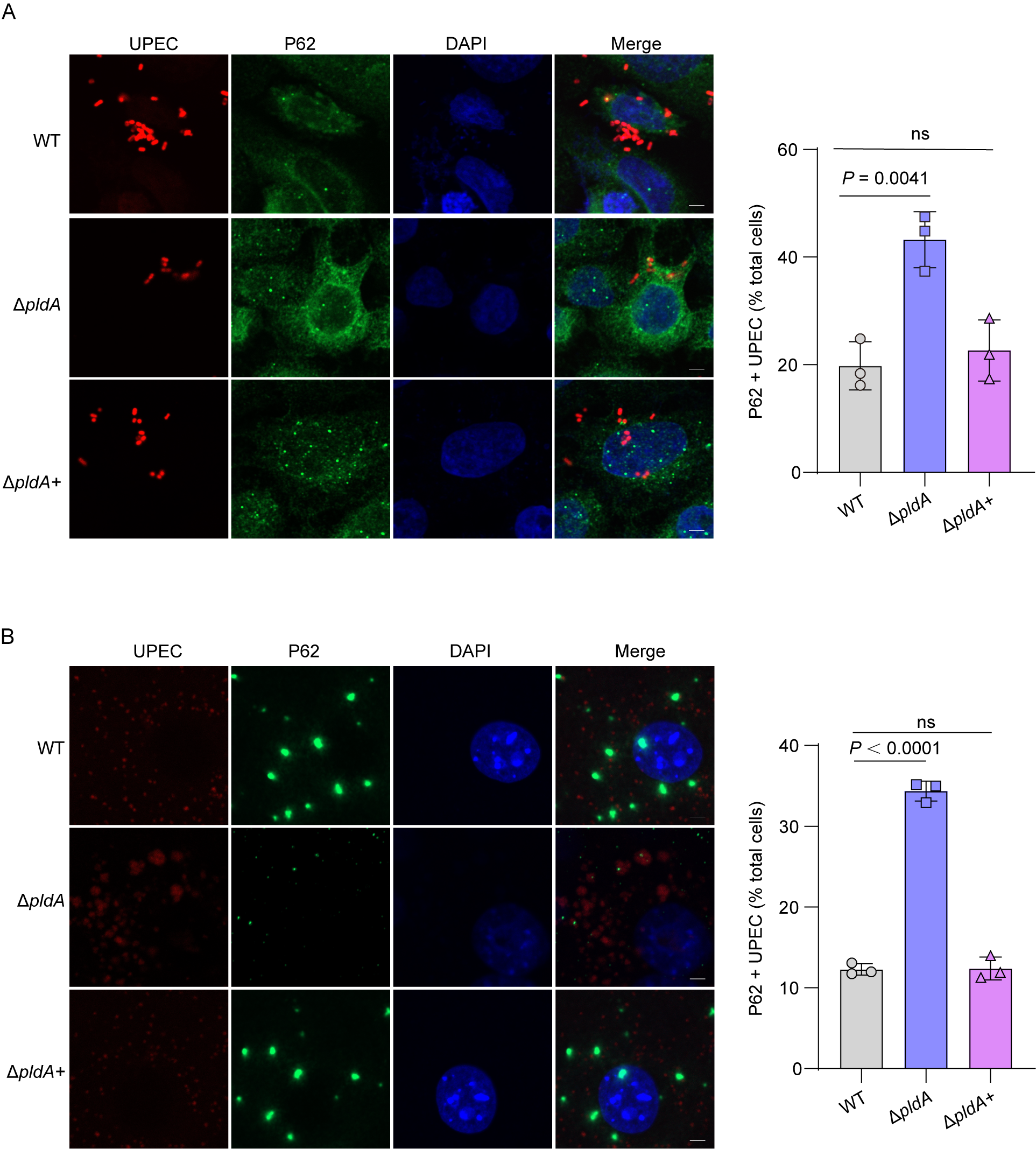

Supplement: jiae063_Supplementary_Data [file jiae063_supplementary_data.zip › SupplementaryFigure 3.tif]

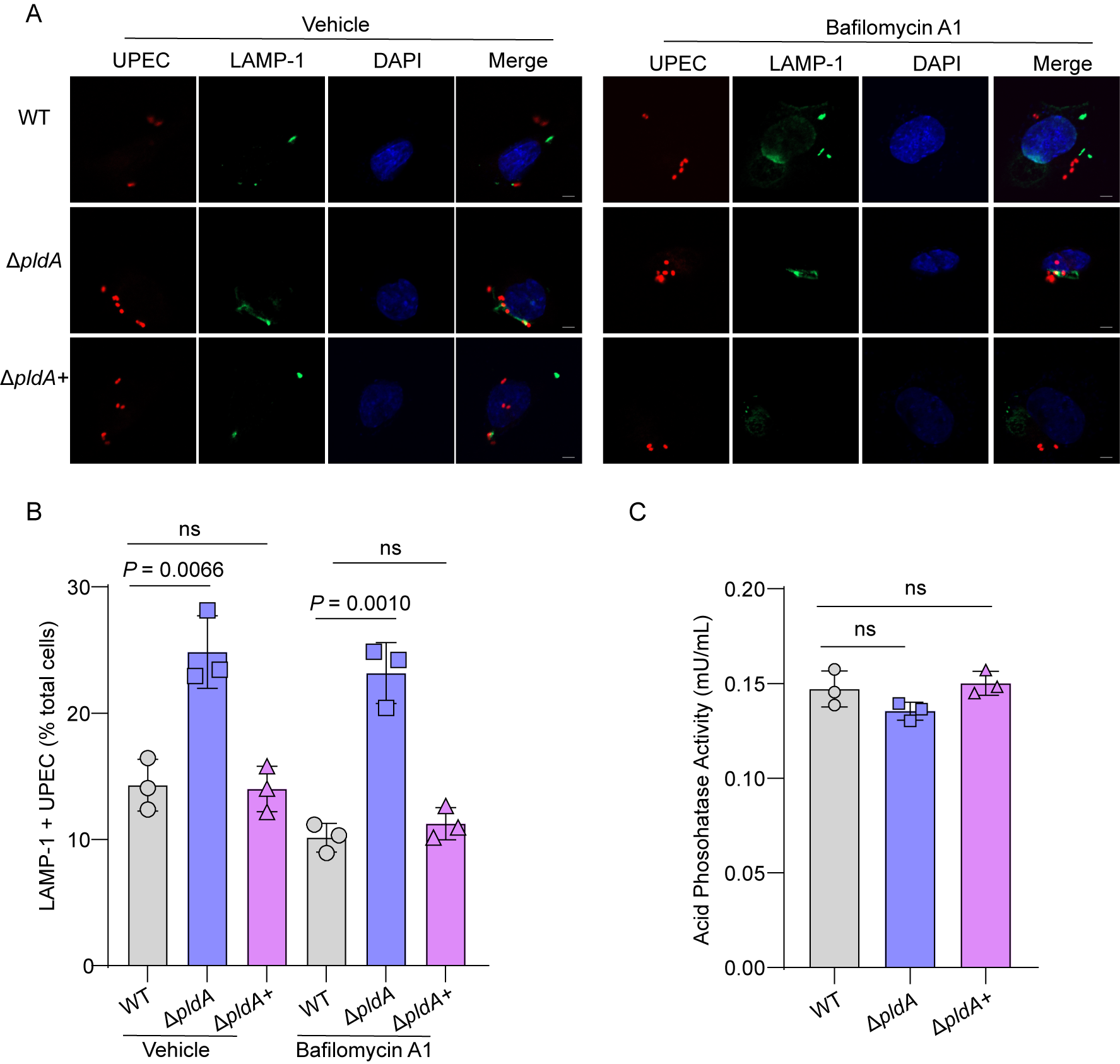

Supplement: jiae063_Supplementary_Data [file jiae063_supplementary_data.zip › SupplementaryFigure 4.tif]

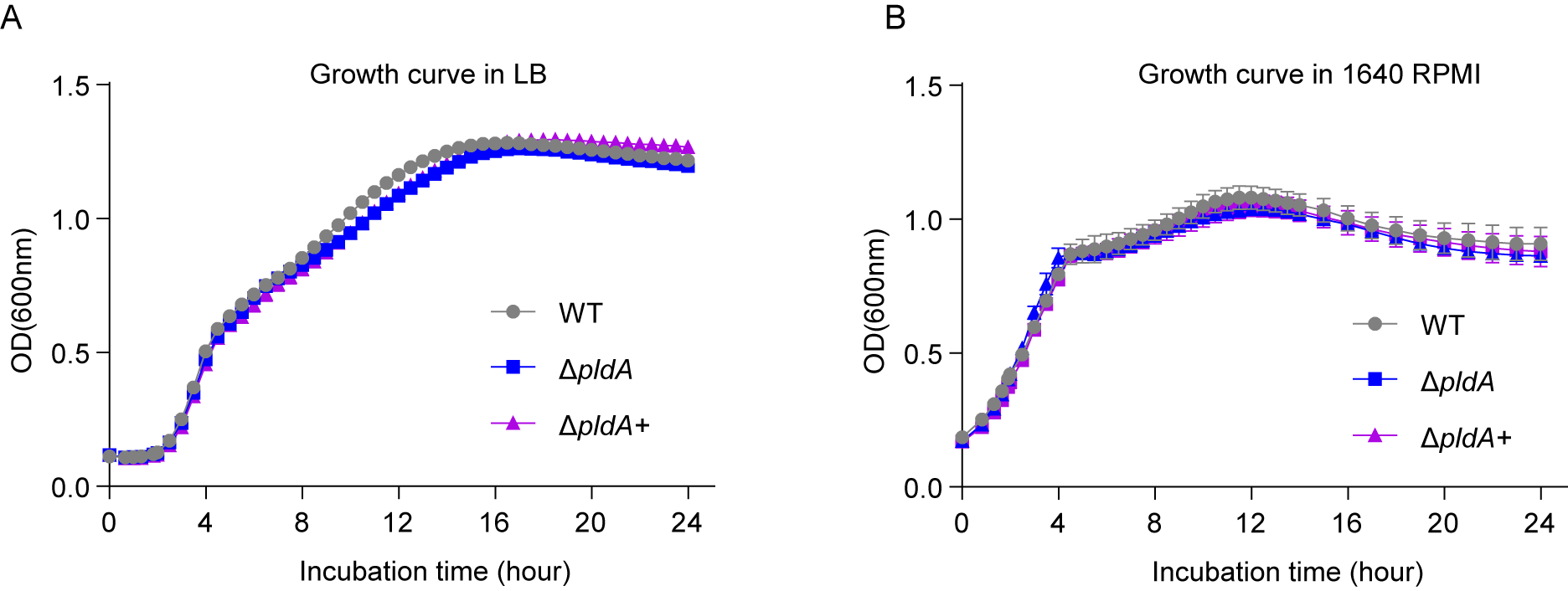

Supplement: jiae063_Supplementary_Data [file jiae063_supplementary_data.zip › SupplementaryFigure 5.tif]
